# Identification of keratinases from *Fervidobacterium islandicum* AW‐1 using dynamic gene expression profiling

**DOI:** 10.1111/1751-7915.13493

**Published:** 2019-10-15

**Authors:** Eunju Kang, Hyeon‐Su Jin, Jae Won La, Jae‐Yoon Sung, Soo‐Young Park, Won‐Chan Kim, Dong‐Woo Lee

**Affiliations:** ^1^ School of Applied Biosciences Kyungpook National University Daegu 41566 South Korea; ^2^ Department of Biotechnology Yonsei University Seoul 03722 South Korea

## Abstract

Keratin degradation is of great interest for converting agro‐industrial waste into bioactive peptides and is directly relevant for understanding the pathogenesis of superficial infections caused by dermatophytes. However, the mechanism of this process remains unclear. Here, we obtained the complete genome sequence of a feather‐degrading, extremely thermophilic bacterium, *Fervidobacterium islandicum* AW‐1 and performed bioinformatics‐based functional annotation. Reverse transcription PCR revealed that 57 putative protease‐encoding genes were differentially expressed in substrate‐dependent manners. Consequently, 16 candidate genes were highly expressed under starvation conditions, when keratin degradation begun. Subsequently, the dynamic expression profiles of these 16 selected genes in response to feathers, as determined via quantitative real‐time PCR, suggested that they included four metalloproteases and two peptidases including an ATP‐dependent serine protease, all of which might act as key players in feather decomposition. Furthermore, *in vitro* keratinolytic assays supported the notion that recombinant enzymes enhanced the decomposition of feathers in the presence of cell extracts. Therefore, our genome‐based systematic and dynamic expression profiling demonstrated that these identified metalloproteases together with two additional peptidases might be primarily associated with the decomposition of native feathers, suggesting that keratin degradation can be achieved via non‐canonical catalysis of several membrane‐associated metalloproteases in cooperation with cytosolic proteases.

## Graphical abstract

Here, we obtained the complete genome sequence (2.23 Mb) of a native feather‐degrading, extremely thermophilic bacterium, Fervidobacterium islandicum AW‐1 and performed bioinformatics‐based functional annotation. The comprehensive analysis of gene expression profiles enabled us to identify keratinolytic proteases from *F. islandicum *AW‐1 at genomic scales. Our genome‐based systematic and dynamic expression profiling suggests that keratin degradation can be achieved via non‐canonical catalytic activities of several membrane‐associated metalloproteases in cooperation with cytosolic proteases.

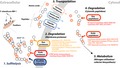

## Introduction

The recent increase in the global consumption of poultry meat has resulted in the ongoing release of a vast amount of native feathers as an environmental solid waste during poultry processing. Crystalline feather keratin is comprised of densely packed β‐pleated sheets that are further stabilized by numerous hydrogen bonds (Suzuki *et al.*, 2006) and disulfide bond cross‐links (Parry *et al.*, 1977). Such insoluble and recalcitrant fibrous proteins (Ashby, 2004) are commonly incinerated, disposed of in landfills, or chemically hydrolysed, resulting in (i) generation of greenhouse gases in the course of waste treatment, concomitant with energy loss; (ii) loss of essential amino acids via acid hydrolysis; or (iii) poor digestibility in the animal gastrointestinal tract with some negative effects on the nutritional value of animal feeds. Although feathers, which are mainly composed of keratin, are potentially useful protein resources for bioactive peptides, rare amino acids, animal feeds and fertilizers (Papadopoulos *et al.*, 1986; Onifade *et al.*, 1998; Gupta and Ramnani, 2006; Jin *et al.*, 2018), their structural rigidity limits their usefulness due to their strong resistance to proteolysis (Parry and North, 1998; Kreplak *et al.*, 2004). The use of keratinases (or microorganisms) as biocatalysts for agricultural and environmental waste treatment to develop environmental recycling technologies for treating keratin‐rich solid waste (Nam *et al.*, 2002; Sharma and Devi, 2018; Yeo *et al.*, 2018; Jin *et al.*, 2019), as well as elucidating the molecular basis of keratin degradation, is especially interesting (Brandelli, 2008). Accordingly, there have been many attempts to identify microbial keratinases due to their attractiveness as tools for the pharmaceutical and cosmetic industries as well as for sustainable waste treatment (Brandelli, 2008; Brandelli *et al.*, 2010; Jin *et al.*, 2018). Despite the identification of several proteases through much effort (Lin *et al.*, 1992; Bockle *et al.*, 1995; Santos *et al.*, 1996; Yeo *et al.*, 2018), their keratinolytic activities have not been fully characterized.

Millions of animals, including humans, around the world are currently suffering from mycosis caused by fungal infections due to inhalation of fungal spores or localized skin colonization under a variety of environmental and physiological conditions (van Baarlen *et al.*, 2007; Revankar and Sutton, 2010). Many infections with fungal pathogens, including organisms belonging to the *Trichophyton*, *Microsporum* and *Epidermophyton* genera, cause dermatophytosis in hair, skin and nails, making them relevant to public human health (Walsh and Groll, 1999; Achterman and White, 2012). Pathogenicity‐associated proteins such as secreted proteases/enzymes are potentially involved in keratin degradation as well as secondary metabolite production (Koziel and Potempa, 2013; Lange *et al.*, 2016). For example, fungal pathogens associated with human disease, such as *Trichophyton rubrum*, *Microsporum gypseum* and *Epidermophyton floccosum*, have keratinolytic enzymes that represent virulence factors involved in superficial and cutaneous dermatophyte infections (Achterman and White, 2012). Indeed, these fungal pathogens can cause severe inflammatory infections in humans, and their genomes contain many protease‐encoding genes relative to phylogenetically related but non‐dermatophytic fungi (Burmester *et al.*, 2011). Secretome analysis of the zoophilic dermatophytes *Arthroderma benhamiae* and *Trichophyton verrucosum* during growth on keratin confirmed that proteases constituted the largest group of identified secreted proteins (Burmester *et al.*, 2011).

Since keratin digestion by the fungus *Onygena equine* was first reported (Ward Harry, 1900), hundreds of keratin‐degrading bacteria and fungi have been isolated and their keratinolytic proteases have been characterized (Monod, 2008); however, the actual key players still remain unclear. It was also noted that combination of several catalytic reactions, including the reduction of disulfide bridges (sulfitolysis), is required for keratin degradation (Bockle and Muller, 1997; Nam *et al.*, 2002; Ramnani and Gupta, 2007). In the light of this, the extremely thermophilic bacterium *F. islandicum* AW‐1, which can degrade chicken feathers completely within 48 h at 70ºC (Nam *et al.*, 2002), would be an excellent model system to investigate the mechanism of keratinolysis under anaerobic conditions, because this bacterium belongs to the order of Thermotogales, which is the most ancient form of bacteria. Intriguingly, our previous LC‐MS/MS analysis revealed that unlike other general serine proteases such as trypsin and proteinase K, *F. islandicum* AW‐1 proteases exhibited a distinct substrate specificity towards feather keratin (Jin *et al.*, 2017). Unlike aerobic superficial dermatophytes such as *M. canis* and *T. rubrum* that secrete the highly conserved set of S3 and M3 endoproteases (Monod, 2008; Sriranganadane *et al.*, 2011), anaerobic *F. islandicum* AW‐1 appears to degrade native feathers via direct cellular adhesion to the substrate by expressing membrane‐bound proteases (Nam *et al.*, 2002). Indeed, this anaerobe did not have any significant extracellular proteases, but whole‐cell extracts including membrane compartment exhibited strong keratinolytic activity (Jin *et al.*, 2017), implying that *F. islandicum* AW‐1 might use an evolutionarily primordial and a different version of keratinolytic machineries under anaerobic conditions. Moreover, despite its minimal genome (Lee *et al.*, 2015b), it has been known as the fastest keratin degrader among the microbial strains reported so far (Gupta and Ramnani, 2006; Brandelli *et al.*, 2010). Therefore, unveiling the *F. islandicum* AW‐1 keratinases would not only provide insight into the degradation mechanism involved in superficial dermatophytosis, but it would also potentiate the development of a biological process in feather waste treatment.

Endo‐ and exo–hydrolases are divided into seven classes (aspartic, cysteine, glutamic, asparagine, serine, threonine and metalloproteases) based on their catalytic mechanism and catalytic residues (Rawlings *et al.*, 2012). Recently, the MEROPS database (DB; http://www.ebi.ac.uk/merops/), which contains a comprehensive list of proteases and their inhibitors, has been developed to provide a classification of proteolytic enzymes along with detailed mechanistic descriptions based on genomic data (Rawlings *et al.*, 2018). In this study, we first obtained the complete *F. islandicum* AW‐1 genome sequence and further validated the functional annotation of its protease‐encoding genes using MEROPS‐based *in silico* analysis. Subsequently, we obtained comprehensive dynamic expression profiles for these genes using reverse transcription (RT) PCR and quantitative real‐time (qRT) PCR to identify keratinases in *F. islandicum* AW‐1. We further used biochemical assays with selected recombinant proteases to determine which enzymes are highly responsive to keratin, when they are expressed, and how they are involved in keratin degradation.

## Results

### The complete *F. islandicum* AW‐1 genome sequence revealed 57 genes encoding putative proteases

To complete the draft genome sequence of *F. islandicum* AW‐1, which consisted of 12 contigs containing 2184 protein‐coding genes (Lee *et al.*, 2015b), we performed next‐generation sequencing (NGS) using P6‐C4 chemistry on the PacBio RS II platform. The raw data for *F. islandicum* AW‐1 yielded 61 549 reads (achieving ~ 397.13‐fold coverage) with an N50 read length of 2 237 377 bp and a mean read length of 11 669 bp, resulting in a single contig of 2 237 377 bp (Fig. S1); these sequence data are more accurate and complete than the previous draft genome sequence. The complete genome (NZ_CP014334.1) revealed that this bacterium contains newly assigned 2065 genes including 1897 protein‐coding genes (Table S1). Whole‐genome comparisons using the ANI calculator (http://enve-omics.ce.gatech) (Rodriguez‐R and Konstantinidis, 2016) revealed average nucleotide identity (ANI) values of 83.69% (*F. islandicum* AW‐1 KCTC 4680^T^, NZ_CP014334.1 versus *F. nodosum* Rt17‐B1, NC_009718.1) and 81.75% (versus *F. pennivorans* DSM9078, NC_017095.1) respectively. Furthermore, eggNOG platform analyses of the *F. islandicum* AW‐1 genome revealed that 1695 of the identified genes could be classified into functional categories based on COG designation (http://www.ezbiocloud.net/) (Fig. S1). *F. islandicum* AW‐1 has two CRISPR‐associated genes that were not identified in the previously sequenced draft genome (Table S1).

To investigate the genomic basis of keratin degradation by proteases and to validate their functional annotations, we classified the 57 genes involved in protein processing and metabolism in the *F. islandicum* AW‐1 genome using the MEROPS DB (Rawlings *et al.*, 2018) and EMBL‐EBI Pfam DB (Table 1). The classification revealed that this bacterium contains functional proteases belonging to six clans (superfamilies) and 31 families, including 30 metalloproteases, 19 serine proteases, two cysteine proteases, two threonine proteases, an aspartic protease and three unidentified proteases (Fig. S2). In particular, the majority of proteases in this bacterium belong to the M48, M42, S14, M23, M50 and S8 families, in that order.

**Table 1:** Functional annotation of putative protease‐encoding genes in *F. islandicum* AW‐1

Locus tag (NA23_)	Protein name	MEROPS ID^b^	Cleavage pattern^c^
RS00455	Ribosomal‐processing cysteine protease Prp	C108.001	−
RS00915	M48 family peptidase	M48.009	−
RS01070^a^	Insulinase family protein	M16.A05 M16.011	− −/−/k/kdl+−/−/−/−
RS01075^a^	ATP‐dependent protease ATPase subunit HslU	T01.006	−
RS01140	Endopeptidase La	S16.001	−/−/−/laf + s/−/−/−
RS01240	ATP‐dependent Clp protease ATP‐binding subunit	S14.001	A/p/−/ml + ay/L/V/P
RS02110	M23 family peptidase	M23.001	−/ayf/−/G + hl/fm/m/−
RS02205	PDZ domain‐containing protein	S01.273 S01.274	−/−/−/V+−/−/−/− −
RS02290	ATP‐dependent metallopeptidase FtsH/Yme1/Tma family protein	M41.001	−/−/−/l+−/−/−/−
RS02410	ATP‐dependent Clp protease ATP‐binding subunit	S14.001	A/p/−/ml + ay/L/V/P
RS03130	M48 family peptidase	M48.009	−
RS03580	Aminopeptidase P family protein	M24.003 M24.004	−/−/−/−+P/−/−/− −
RS03770	Aminopeptidase	M18.001	−
RS04220	Carboxypeptidase M32	M32.001	−
RS04280	M42 family peptidase	M42.001 M42.002 M42.006	− − −
RS04555	Signal peptidase I	S26.001	−/A/−/A + a/ed/−/−
RS04625^a^	Aminopeptidase	M29.001	−
RS04640^a^	NfeD family protein	S49.005	−
RS04740	ATP‐dependent protease subunit HslV	T01.006	−
RS05345^a^	ATP‐dependent Clp protease ATP‐binding subunit ClpX	S14.001	A/p/−/ml + ay/L/V/P
RS05350^a^	tRNA (adenosine(37)‐N6)‐threonylcarbamoyltransferase complex transferase subunit TsaD	M22	−
RS05465	DUF1751 domain‐containing protein	S54.016	−
RS05565	Pyroglutamyl‐peptidase I	C15.001	−
RS05775	Peptidase S8	S08.007	−/−/a/−+−/−/−/−
RS06095	Alpha/beta hydrolase	S09.001 S09.A47	p/pq/−/P + q/Pg/q/pl −
RS06130	Dipeptidase PepV	M20.004	−
RS06425	Prepilin peptidase	A24.001	ar/Q/krs/G + F/T/L/IL
RS06435	Serine protease	S01.274	−
RS06495	Peptidase	M50.004	−
RS06750	Peptidase S8	S08.021	−
RS06760	M42 family peptidase	M42.001 M42.002 M42.006	− − −
RS06770	M42 family peptidase	M42.001 M42.002 M42.006	− − −
RS06815	Peptidase S8	S08.021	−
RS06975^a^	ATP‐dependent Clp endopeptidase proteolytic subunit ClpP	S14.001	A/p/−/ml + ay/L/V/P
RS07020^a^	Type I methionyl aminopeptidase	M24.001	−/−/−/M + sat/−/−/−
RS07390	Site‐2 protease family protein	M50.001 M50.002	− −
RS07510	tRNA (adenosine(37)‐N6)‐threonylcarbamoyltransferase complex dimerization subunit type 1 TsaB	‐	−
RS07665	Peptidase M3	M03.007	−/−/−/l + g/p/l/−
RS07735	M15 family peptidase	M15.010	−
RS07755	S9 family peptidase	S09.001	p/pq/−/P + q/Pg/q/pl
RS07775	M15 family peptidase	M15.010	−
RS07975	Peptidase	M50.A11	−
RS08100	β‐aspartyl‐peptidase	M38.001	−
RS08155	D‐alanyl‐D‐alanine carboxypeptidase	S13.001	−
RS08385	M48 family peptidase	M48.003	−
RS08750	CPBP family intramembrane metalloprotease	M48.003	−
RS09140	Hypothetical protein	M48.002	−
RS09535	Rhomboid family intramembrane serine protease	S54.016	−
RS09555	Peptidase M3	M03.007	−/−/−/l + g/p/l/−
RS09565^a^	TldD/PmbA family protein	U62.002	−
RS09570^a^	TldD/PmbA family protein	U62.002	−
RS09715	M42 family peptidase	M42.001 M42.002 M42.006	− − −
RS09765	S41 family peptidase	S41.013 S41.004	− −
RS09910^a^	ATP‐dependent protease	S16.005	−
RS09930^a^	M23 family peptidase	M23.001	−/ayf/−/G + hl/fm/m/−
RS10350	Peptidase M23	M23.950	−
RS10455	Peptidase M55	M55.001	−

a Predicted in the operon structure using DOOR analysis (http://csbl.bmb.uga.edu/DOOR/index.php) (Mao *et al.*, 2008).

b A, aspartic; C, cysteine; M, metalloproteases; S, serine; T, threonine; U, proteases with unknown catalytic mechanism.

c One‐letter code, the preferred amino acid residues in each site; plain text with oblique strokes, cleavage pattern and P4‐P4' positions; larger‐ and smaller‐case letters, the strength of the specificity preference; +, scissile bond.

### Depletion of soluble nutrients triggered feather degradation by *F. islandicum* AW‐1

**Figure 1:**
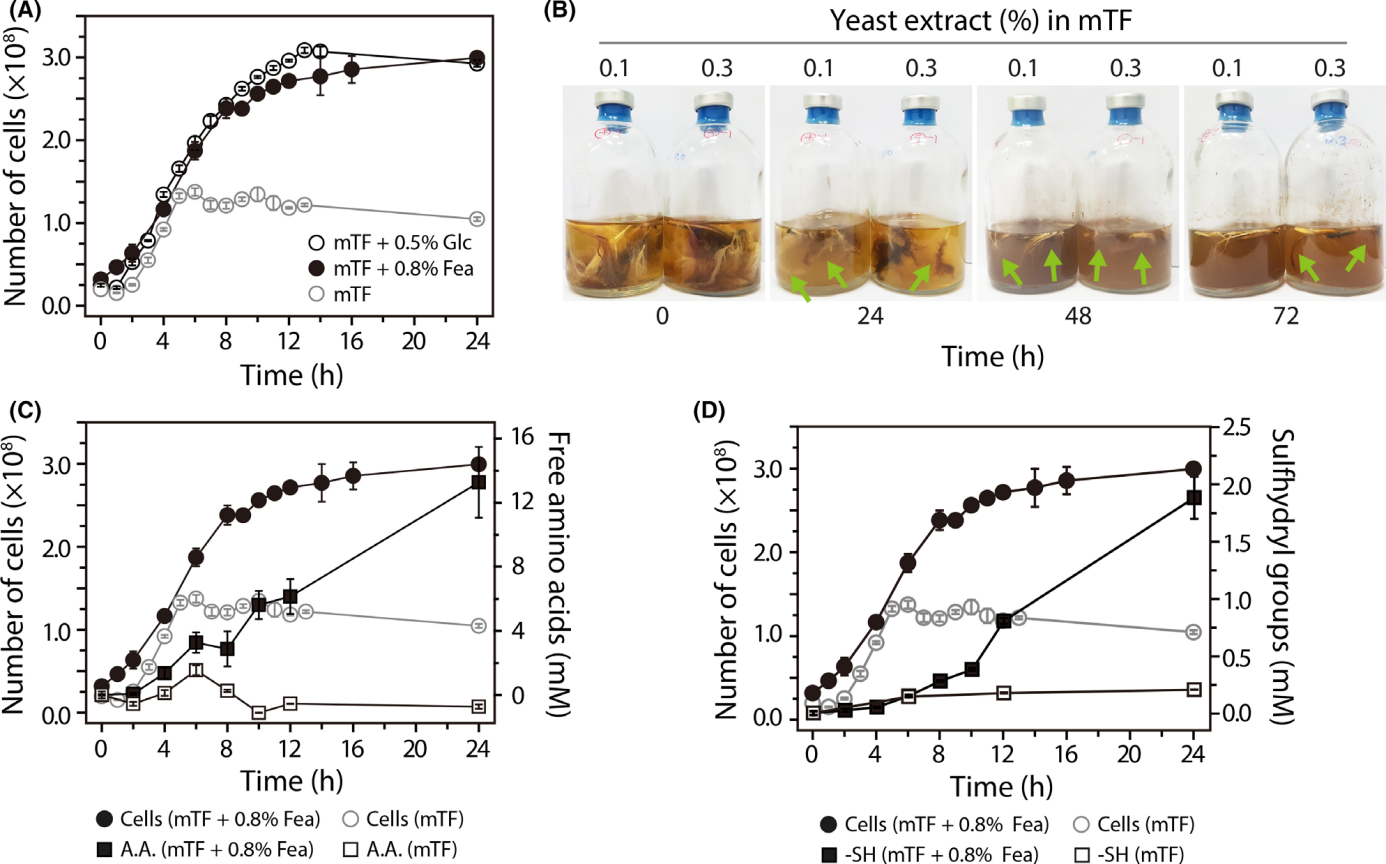
Time‐courses of *F. islandicum* AW‐1 growth and degradation product accumulation on different nutrients. A. Growth curves of *F. islandicum* AW‐1 in mTF medium only and mTF medium supplemented with 0.5% (w/v) glucose or 0.8% native chicken feathers during anaerobic cultivation at 70°C. For the seed culture, bacteria were grown on mTF medium (50 ml) containing 5% glucose at 70°C for 12 h. The inoculum for the liquid medium was prepared by growing *F. islandicum* AW‐1 to late exponential phase (approximately 2 × 10^8^ cells ml^−1^). B. Complete degradation of native feathers by *F. islandicum* AW‐1 grown in mTF medium containing 0.1% (left) or 0.3% (right) yeast extract. Arrows coloured in green indicate the feather degradation occurring regions. Time‐course of (C) free amino acid production and (D) sulfhydryl group formation from feather degradation by *F. islandicum* AW‐1 grown in mTF medium containing 0.1% yeast extract supplemented with or without 0.8% feathers. Free amino acid production was assessed using the ninhydrin method (Rosen, 1957), and the sulfhydryl groups were detected using the Ellman method (Ellman, 1959).

To investigate the growth physiology of the native feather‐degrading, extremely thermophilic anaerobe *F. islandicum* AW‐1, we first obtained the growth profiles of this bacterium when grown at 70°C in mTF medium containing 0.1% yeast extract only and in media supplemented with either 0.5% (w/v) glucose or 0.8% (w/v) feathers (Fig. 1A). The *F. islandicum* AW‐1 cells in the mTF medium supplemented with 0.5% glucose or 0.8% feathers reached 3 × 10^8^ cells ml^−1^ after 12 h of incubation. On the other hand, cells grown in mTF only reached 1.2–1.3 × 10^8^ cells^−1^ml^−1^ within 5 h, after which no further growth was observed. Subsequently, we added 0.3% yeast extract to the mTF medium to investigate the effect of yeast extract on feather degradation by *F. islandicum* AW‐1. Feather degradation was observed within 24 h when the cells were grown in mTF containing 0.1% yeast extract, whereas cells in mTF with 0.3% yeast extract exhibited feather degradation after 24 h (Fig. 1B). These observations demonstrated that a relatively small increase in the amount of yeast extract might delay the decomposition of native feathers, indicating that *F. islandicum* AW‐1 prefers to use yeast extract as a nutrient source rather than native feathers.

To further validate this hypothesis, we determined the amounts of free amino acids and sulfhydryl groups formed during feather hydrolysis in the culture broth during the course of bacterial growth. The free amino acid production by cells grown in the mTF only slightly increased over 5 h of incubation and then decreased during an additional 3 h of incubation (Fig. 1C). However, the broth containing cells grown in mTF showed no increase in sulfhydryl group abundance even after 24 h (Fig. 1D). On the other hand, cells grown in mTF supplemented with 0.8% feathers produced not only free amino acids, but also sulfhydryl groups during the course of bacterial growths, indicating that *F. islandicum* AW‐1 utilized the native feathers to gain nutrients for its growth. Both the free amino acid and sulfhydryl production profiles were biphasic during the course of bacterial growth in mTF with 0.8% feathers. The amino acid production rate was decreased at around 6 h, when the yeast extract was depleted (Fig. 1C). On the other hand, sulfhydryl groups were not detected until 6 h of incubation, but their abundance significantly increased thereafter (Fig. 1D). Taken together, these results indicated that feather decomposition by *F. islandicum* AW‐1 occurred after depletion of the yeast extract, suggesting that feather degradation is triggered upon depletion of soluble nutrients when only insoluble feathers as substrates are available. Therefore, it is anticipated that feather‐degrading enzymes except a few constitutively expressed enzymes should be highly expressed in the presence of feathers.

### Differential expression profiles of protease‐encoding genes revealed protease partitioning in substrate‐ and growth phase‐dependent manners

To systematically unveil the key player(s) responsible for feather degradation, we used RT‐PCR to investigate the expression levels of 57 putative protease‐encoding genes in cells grown on feathers versus glucose at different growth stages. To this end, we first collected cells grown on glucose or feathers at time points before (approximately 1.2 × 10^8^ cells ml^−1^) and after (approximately 1.6 × 10^8^ cells ml^−1^) depletion of the yeast extract as a supply of basal nutrients (Fig. 1A). Thereafter, we synthesized cDNAs from the total RNA extracted from the collected cells to compare the expression patterns of those genes using RT‐PCR to determine which genes are more highly expressed in cells grown with feathers rather than glucose as their carbon nutrient. As shown in Fig. S3, it was observed that not only did the expression patterns of the genes vary depending on the provided nutrient source, but also that different sets of genes were highly expressed or downregulated before and after yeast extract depletion. Consequently, out of the 57 putative protease‐encoding genes from *F. islandicum* AW‐1, 21 genes were expressed in the presence of nitrogen sources, including feathers. The genes expressed before yeast extract depletion were considered general proteases involved in nitrogen metabolism, whereas the remaining 16 proteases might play important roles in feather degradation.

**Figure 2:**
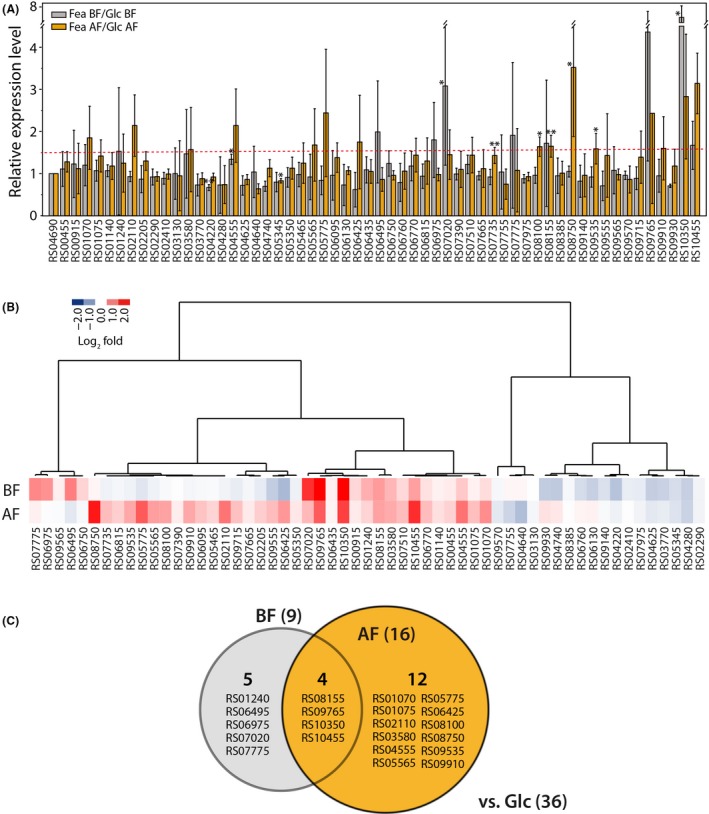
Growth phase‐ and nutrient‐dependent expression levels of protease‐encoding genes assessed via reverse transcription (RT) PCR. A. Relative expression levels of the *F. islandicum* AW‐1 protease‐encoding genes. Total mRNA was obtained from cells before (BF) and after (AF) the yeast extract was depleted in mTF medium supplemented with 0.5% (w/v) glucose (Glc) or 0.8% native chicken feathers (Fea) during anaerobic fermentation at 70°C (as described above). The RT‐PCR data quantified by measuring the band intensity using ImageJ. The σ‐70 RNA polymerase subunit was used as positive control for expression. *P < 0.05 and **P < 0.01 (paired *t*‐test) compared with the mRNA expression level in cells grown on glucose. B. Heat‐map diagram of a hierarchical clustering analysis consisting of the 57 differentially expressed protease‐encoding genes. C. Venn diagram of the genes differentially expressed in bacteria grown on feathers compared with those in bacteria grown on glucose.

Indeed, quantitative analysis by band intensity‐based image analysis revealed that the expression levels of 21 genes were more than 1.5 times higher when the cells were grown on feathers than when they were grown on glucose (Fig. 2A and B). Nevertheless, we could not exclude the possibility that some of these genes encode proteins primarily used to gain nitrous nutrients from the yeast extract to support bacterial growth independent of feather decomposition (Fig. 1A). In this case, those genes could introduce noise into a screen for genes that are highly responsive to feathers. Accordingly, we primarily focused on the genes with more than 1.5‐fold greater expression in cells grown on feathers than in cells grown on glucose after yeast extract depletion in the mTF medium (approximately 1.6 × 10^8^ cells ml^−1^). This analysis indicated that 16 genes (i.e. RS01070, RS01075, RS02110, RS03580, RS04555, RS05565, RS05775, RS06425, RS08100, RS08155, RS08750, RS09535, RS09765, RS09910, RS10350 and RS10455) might serve as potential feather decomposers (Fig. 2). Taken together, we tentatively concluded that 21 protease‐coding genes might play major roles in nitrogen source utilization (Fig. 2C). Furthermore, we anticipated that, out of these genes, the 16 proteases highly expressed after yeast extract depletion might play important roles in feather degradation.

### Dynamic expression profiling of protease‐encoding genes identified a distinct set of proteases responsible for keratin degradation

**Figure 3:**
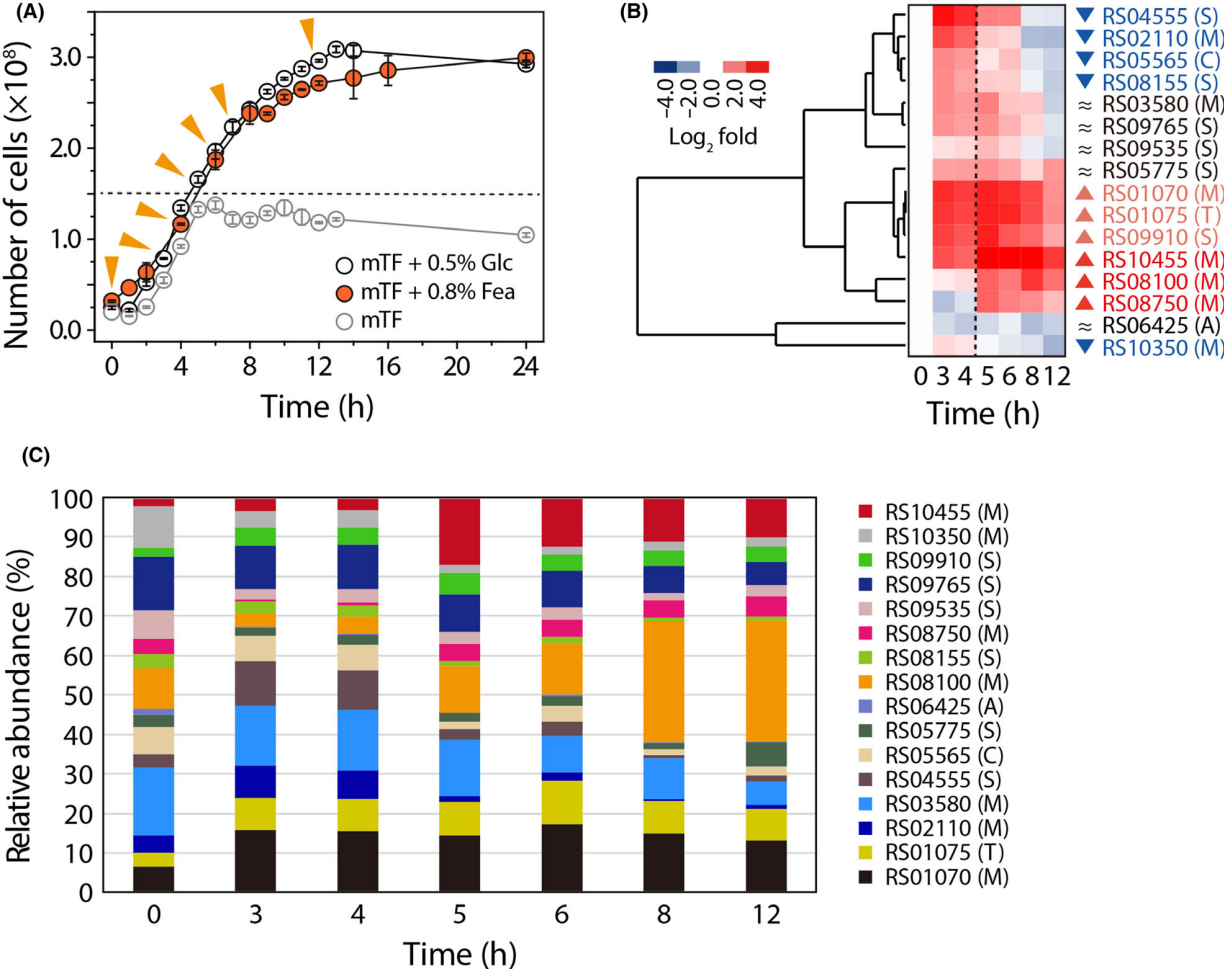
Dynamic expression profiling of putative keratinase‐encoding genes in *F. islandicum* AW‐1. A. Growth curves of *F. islandicum* AW‐1 in mTF medium only and mTF medium supplemented with 0.5% (w/v) glucose or 0.8% native chicken feathers during anaerobic cultivation at 70°C. Triangle symbols (yellow) indicate the collection time points for mRNA preparation. B. Heat‐map diagram of a hierarchical clustering analysis consisting of the selected 16 differentially expressed protease‐coding genes. Quantitative real‐time (qRT) PCR analysis was performed to investigate the dynamic expression profiles of 16 selected genes in detail. For qRT‐PCR, cells were grown in mTF medium supplemented with 0.8% chicken feathers. The mRNA quality was assessed via electrophoretic analysis of the rRNA and measurement of the absorbance at 260 nm. The σ‐70 RNA polymerase subunit was used as a positive control for expression. The expression levels of the individual genes at the different time points were compared with those of the corresponding genes at 0 h. C. Relative abundance profiles of 16 differentially expressed proteases in *F. islandicum* AW‐1.

To further define the expression levels of the 16 protease‐encoding genes specifically involved in feather degradation, we used qRT‐PCR analysis to obtain the time‐dependent expression profiles over the course of seven different growth phases (i.e. early, mid‐ and late exponential and stationary phases, and the time points before and after depletion of yeast extract as a nutrient source) (Fig. 3A). The growth phase‐ and nutrient‐dependent expression profiles of these genes clearly indicated that three different sets of genes could be defined with respect to their expression patterns (Fig. 3B). This observation revealed (i) one set of genes whose expression levels are constant regardless of whether or not the yeast extract is depleted (i.e. RS03580, RS05775, RS06425, RS09535 and RS09765), (ii) a set of genes whose expression levels are high before yeast extract depletion and then decrease over time (RS02110, RS04555, RS05565, RS08155 and RS10350), and (iii) a set of genes that are highly expressed after yeast extract depletion (RS01070, RS01075, RS08100, RS08750, RS09910 and RS10455). Furthermore, we also assessed the relative abundance of each specific gene relative to the combined expression of the proteases at each time point designated above (Fig. 3C). Remarkably, the genes encoding several metalloproteases, including RS08100, RS08750 and RS10455, were suppressed until yeast extract depletion but were then significantly expressed after its depletion. Notably, these highly expressed metalloprotease genes accounted for more than 60% of the total abundance of expressed proteases, indicating that these enzymes are highly abundant and specific to feather degradation. Furthermore, several constitutively expressed proteases, including RS03580, RS05775, RS09535 and RS09765, also appeared to be involved in feather degradation. Therefore, these results clearly indicated that a group of proteases is constitutively expressed to sustain cellular metabolism in the absence of nitrogen sources, whereas another group including mainly metalloproteases is highly responsive to the presence of feathers as a nutrient source. The third group is mainly involved in amino acid transport and metabolism involving nitrous resources within cells. Previously, we observed that crude extracts from *F. islandicum* AW‐1 cells showed little activity for α‐keratinous substrates such as hairs and nails (unpublished data). Taken together, we suggest that *F. islandicum* AW‐1 can decompose native feathers via keratinases consisting of feather‐specific metalloproteases and constitutively expressed proteases.

### Several metalloproteases, including a few serine proteases, are major contributors to native chicken feather degradation

**Figure 4:**
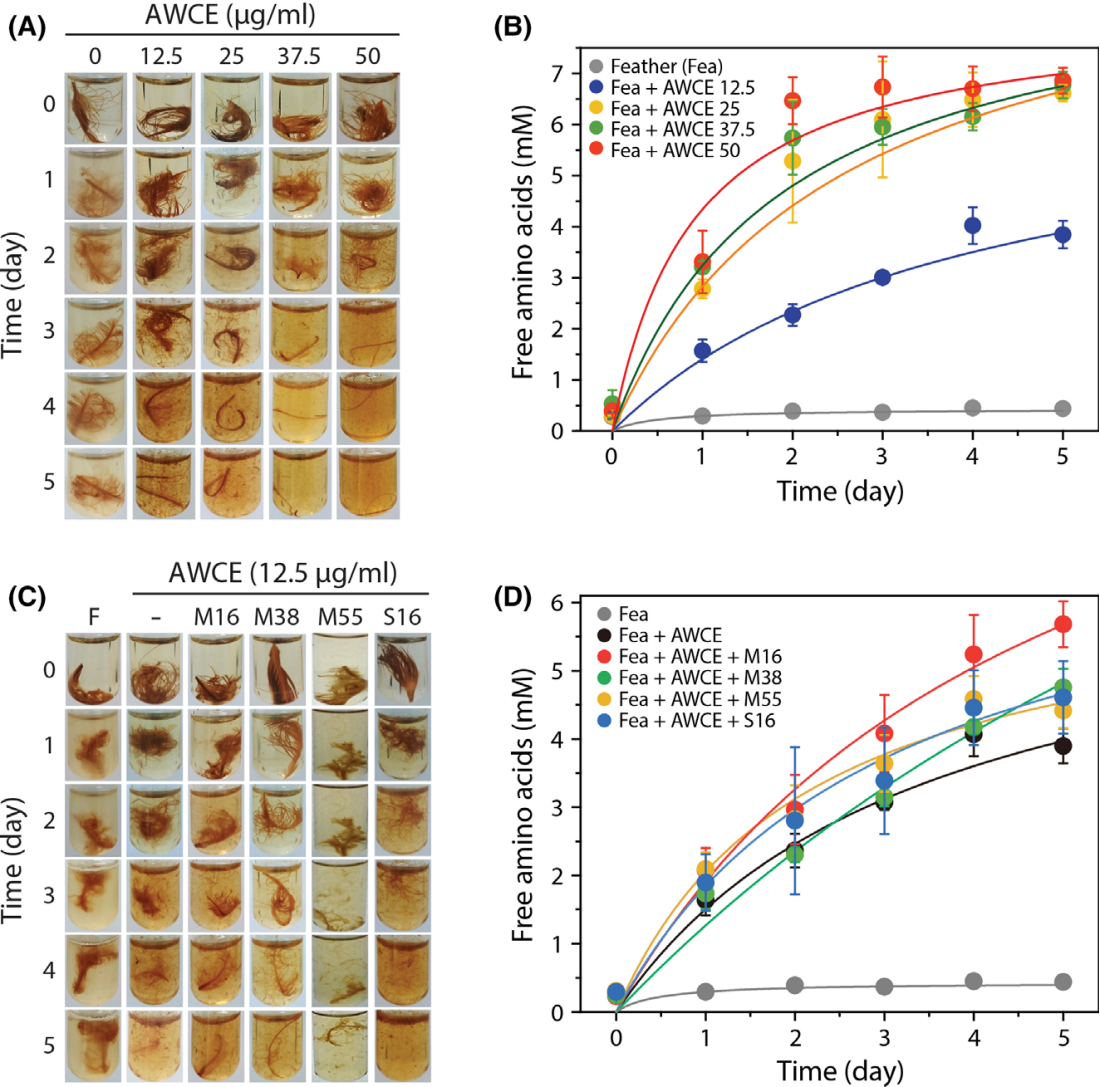
The effects of recombinant keratinases on native feather degradation. A. Photographs of native feather degradation by AWCE at different concentrations (0 to 50 µg ml^−1^) in the presence of 10 mM DTT. B. Time‐course of free amino acid production during degradation of native feathers by AWCE at different concentrations (0 to 50 µg ml^−1^) in the presence of 10 mM DTT. C. Photographs of native feather degradation by each enzyme (0.15 mg) in the presence of AWCE (0.0375 mg) and 10 mM DTT at 72°C. F, feather; M16, insulinase family protein (encoded by RS01070); M38, β‐aspartyl‐peptidase (RS08100); M55, peptidase M55 (RS10455); S16, ATP‐dependent protease (RS09910). D. Time‐course of free amino acid production during degradation of native feathers by each enzyme (0.15 mg).

Based on the expression profiles and relative abundance analysis of the expressed genes, we chose six putative keratinase‐encoding genes (i.e. RS01070, RS01075, RS08100, RS08750, RS09910 and RS10455) to express in *E. coli* BL21 (DE3) for purification of recombinant proteins using Ni^2+^‐affinity chromatography following heat treatment at 70°C (Table S4 and Fig. S4). Out of the six genes, four were successfully expressed as soluble proteins (Fig. S4A). To investigate whether these proteases can enhance the *in vitro* feather degradation activity described previously (Lee *et al.*, 2015a; Jin *et al.*, 2017), purified recombinant enzymes were added to crude *F. islandicum* AW‐1 extract (AWCE) from glucose‐grown cells containing basal levels of proteases, which was then incubated with 0.2% (w/v) native feathers in the presence of 10 mM DTT under anaerobic conditions at 72°C (Fig. 4 and Fig. S4B). Prior to this, we first investigated the effect of AWCE concentration ranging from 0 to 50 µg ml^−1^ on feather degradation (Fig. 4A and B). As a result, the rate of feather decomposition was positively correlated with the amount of AWCE used, and the minimal level of AWCE containing 12.5 µg ml^−1^ total protein was determined for monitoring the additional effect of purified enzymes. The AWCE (12.5 µg ml^−1^) alone exhibited sufficient feather‐degrading activity to completely degrade native feathers in 5 days, resulting in the production of 4 mM free amino acids (Fig. 4B). Remarkably, addition of each purified enzyme to the AWCE reaction mixture accelerated feather decomposition with increased free amino acid yields (Fig. 4C and D). Moreover, the free amino acid production rates following addition of purified enzymes to each reaction mixture were, on average, 1.5‐fold higher than those of AWCE only, suggesting that three identified metalloproteases, including β‐aspartyl‐peptidase (M38), peptidase M55 and an insulinase family protein (M16) together with an ATP‐dependent serine protease S16, robustly degrade native feathers as substrates at elevated temperatures.

## Discussion

In this study, we mainly focused on the identification of hitherto unknown keratinases in this anaerobic bacterium by a comprehensive screening of proteases based on their dynamic expression profiles and on genome information, thereby providing a list of potential keratin‐specific proteases (Figs S1 and S3 and Table 1). Together with a fundamental question whether the microorganism decomposes native keratin to utilize a nutrient source during their growth or other minor non‐keratinous constituents (Noval and Nickerson, 1959), another important issue can be raised regarding substrate preference between soluble and insoluble nutrients in relation to keratin digestion. Previously, superficial dermatophyte *M. canis* secreted different proteases depending on the growth medium (carbon versus nitrogen sources) and the infectious process (Brouta *et al.*, 2002). Indeed, the growth phase‐dependent gene expression patterns of *F. islandicum* AW‐1 revealed that of 57 putative protease‐encoding genes, 21 genes were expressed more robustly in cells grown on feathers than in cells grown on glucose (Fig. S3); however, the expression levels of several genes were marginal probably due to the presence of yeast extract in the mTF medium containing feathers. The availability of yeast extract retarded sulfhydryl group release from feather degradation, indicating that, under such conditions, soluble nitrogen sources are the primary nutrient for *F. islandicum* AW‐1 cells rather than insoluble feather keratin (Fig. 1). Therefore, we further compared the mRNA expression levels of those genes before and after yeast extract depletion to yield a set of 11 potential proteases involved in keratin degradation (Fig. 2). With the exception of three enzymes, these proteases can be grouped into the metalloprotease and serine protease families (Fig. S2). The expression of proteolytic enzymes, including *Bacillus* keratinases (subtilisin‐like serine protease, S8 family), is highly dependent on growth phase and nutrient availability (Kumar and Takagi, 1999), and most of them are particularly abundant in late exponential or stationary phase when the levels of nutrients or other nutritional factors (e.g. carbon, nitrogen or phosphate) are limited or exhausted (Voigt *et al.*, 2007; Daroit and Brandelli, 2014). Consistently, the expression patterns and levels of these 16 protease‐encoding genes in *F. islandicum* AW‐1 analysed by qRT‐PCR revealed that, upon yeast extract depletion, six genes (i.e. RS01070, RS01075, RS08100, RS08750, RS09910 and RS10455) were significantly expressed when feathers were provided as the sole nutrient (Fig. 3A and B). The relative abundance of their gene expression levels supports the notion that these six proteases were mainly involved in keratin degradation (Fig. 3C). Notably, these six potential keratin‐specific proteases in *F. islandicum* AW‐1 (i.e. proteases in M16, T01, M38, M48, S16 and M55 families) are different from keratinases (e.g. proteases in S8, M28 and M3 families) in superficial dermatophytes (Monod, 2008).

To validate the keratinolytic activity of these proteases *in vitro*, we successfully expressed four of the proteases in soluble forms in *E. coli* (Fig. S4). When added to reaction mixtures containing WCEs derived from *F. islandicum* AW‐1 cells, these enzymes accelerated and/or enhanced the degradation of native chicken feathers, resulting in the release of free amino acids from feather keratin (Fig. 4), which was in accordance with the previous results (Lee *et al.*, 2015a; Jin *et al.*, 2017). It could be speculated that peptide fragments produced from feathers by activated cytoplasmic endo‐ and exoproteases might act as transcription triggering factors or signalling molecules via peptide transporters to promote feather decomposition and utilization. Although the biological functions of these proteases remain unclear, it is evident that these enzymes play an important role in the keratin degradation in cooperation with proteins encoded by a group of genes also involved in feather degradation, but which have constitutively high expression levels regardless of yeast extract depletion (i.e. RS03580 and RS09765). Overall, these results suggest that feather decomposition cannot be achieved by a protease, but it would rather require a combination of multiple enzymes, including unknown sulfitolytic components. Such a synergism for keratin degradation was also supported by several recent studies on keratin degradation by dermatophytes provided a plausible mechanism that pathogenic dermatophytes cleave disulfide bonds excreting the reducing agent sulfite through cysteine dioxygenase and sulfite efflux pump (Grumbt *et al.*, 2013), and then degrade keratin peptides using a combination of several proteases, including endoproteases, exoproteases and oligopeptidases (the S8, M28 and M3 families respectively), and contribute to keratin decomposition (Huang *et al.*, 2015).

**Figure 5:**
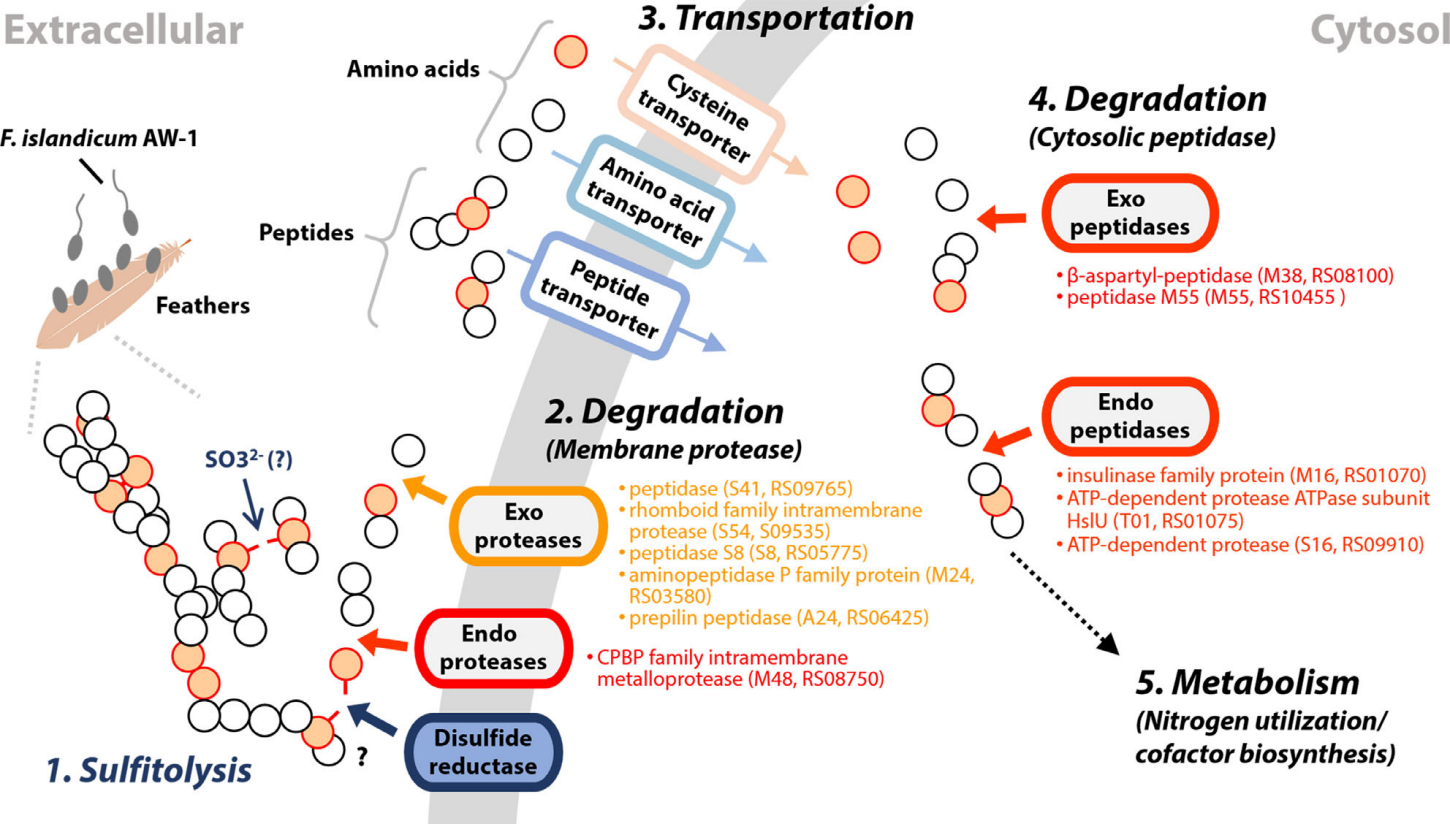
Proposed mechanism of feather degradation by *F. islandicum* AW‐1. MEROPS‐based functional annotation of the protease‐encoding genes and their expression patterns suggest that either sulfite or unidentified disulfide reductase may be involved in cleavage of disulfide bonds (Group 1, sulfitolytic machinery) and that the CPBP family intramembrane metalloprotease (RS08750) and additional five membrane proteases (RS03580, RS05775, RS06425, RS09535 and RS09765) act as key keratinases (Group 2, degrader). Subsequently, peptidase M55 (RS10455) and the β‐aspartyl‐peptidase (RS08100) may be associated with decomposition of the peptide into free amino acids as signals (Group 4, degrader). In addition, the insulinase family protein (encoded by RS01070) and ATP‐dependent proteases (RS01075 and RS09910) may be involved in the regulation of proteolysis during feather degradation (Group 4, degrader). Upon yeast extract depletion, highly expressed genes are coloured in red and boldface, and constitutively expressed genes regardless of yeast extract depletion are coloured in pink. Circles depict cysteine (pale orange) and other amino acids (white).

Based on the *in silico* functional annotation and expression patterns of the protein‐encoding genes in this study, it is possible to predict which types of enzymes (i.e. endo‐ and exoproteases) degrade keratin, where those enzymes are located, and when they are expressed in keratin degradation (Fig. 5). First, the insulinase (peptidase M16) family protein (RS01070), ATP‐dependent protease ATPase subunit HslU (RS01075), M24 aminopeptidase P family protein (RS03580), β‐aspartyl‐peptidase M38 (RS08100), the CAAX proteases and bacteriocin‐processing protein (CPBP) family intramembrane metalloprotease (RS08750), S41 family peptidase (RS09765), S16 ATP‐dependent protease (RS09910) and peptidase M55 (RS10455), which were highly expressed and abundant in cells grown on feathers (Fig. 3C), might be directly involved in feather decomposition, although their exact biological functions remain to be established. Based on the polycistronic organization of RS01070 and RS01075 in the genome (the initiation of RS01075 is positioned immediately downstream of RS01070), the two genes could be co‐transcribed and might function as similar counterparts to major exopeptidases such as Lap1 and Lap2 in *T. rubrum* (Monod, 2008). Out of these proteases described above, β‐aspartyl‐peptidase M38, the endotype CPBP family intramembrane metalloprotease presumably responsible for regulated proteolysis (Pei *et al.*, 2011), and the peptidase M55 (RS10455), a D‐aminopeptidase dipeptide‐binding protein family (IPR007035) belonging to the dipeptide ABC transport (dpp) operon, function as an adaptation to nutrient deficiency (Remaut *et al.*, 2001), were significantly upregulated upon yeast extract depletion (Fig. 3). The M55 homologue DppA in *B. subtilis*, which belongs to the dipeptide ABC transport operon, is expressed early during sporulation and under nutrient‐depleted conditions (Cheggour *et al.*, 2000). Therefore, these three enzymes might be highly responsive to starvation conditions and involved in regulation of peptides as degradation products. In addition, the constitutively expressed five proteases (i.e. RS03580, RS05775, RS06425, RS09535 and RS09765) might share similar features to dermatophyte‐secreted exopeptidases such as leucine aminopeptidases and dipeptidyl‐peptidases in *T. rubrum* (Monod, 2008). Various metalloproteases, including those in the M14 metalloprotease family (Riffel *et al.*, 2007), have been reported as keratin decomposers (Allpress *et al.*, 2002; Thys and Brandelli, 2006; Balaji *et al.*, 2008; Lin *et al.*, 2009). These results are consistent with those of a previous study in which DppIV, a predicted virulence factor in dermatophytes, was identified in *M. canis* based on sequence similarity (Gräser *et al.*, 1999). Notably, constitutively expressed proteases such as rhomboid domain‐containing membrane protease S54 (RS09535) highly conserved in three kingdoms of life, c‐terminal exopeptidase S41 (RS09765) and subtilisin‐related S8 protease (RS05775) might contribute to utilization of keratin peptides as nitrogen sources. ATP‐dependent protease (RS01075) exhibits high levels of similarity with a member of the Hsp100 (Clp) proteases (Bochtler *et al.*, 2000). Consequently, the resulting keratin hydrolysates produced by the above enzymes are further processed by intracellular proteases to support cell growth, including Fe‐S cluster biosynthesis (Fig. 5). However, it has not yet been clarified which enzymes and/or chemicals such as sulfite facilitate disulfide bond cleavage and which enzymes help to unwind the keratin structure.

Currently, a comprehensive functional genomics analysis using a gene knockout system is underway to further characterize the proteases identified in this study. Such data may further strengthen our understanding of the molecular mechanism of keratin digestion, which underlies the pathogenesis of superficial dermatophyte infections. Furthermore, these enzymes can be exploited to design and develop an environmentally favourable and efficient recycling process to treat solid keratinous waste, which was supported by recent works highlighting the power of keratin hydrolysates as bioactive peptides in pharmaceuticals and cosmetics (Villa *et al.*, 2013; Hou *et al.*, 2017; Jin *et al.*, 2018).

## Experimental procedures

### Ethics statement

The chicken feathers used in this study were obtained from a medium‐sized poultry production unit in Kyungpook Province. This study does not belong to the animal studies authorized by the Institutional Animal Care and Use Committee of Kyungpook National University and Yonsei University. No approval from an ethical committee was required for this study.

### Anaerobic culture techniques

*Fervidobacterium islandicum* AW‐1 from the Korean Collection for Type Cultures (KCTC) with the accession number KCTC 4680 was grown in modified *Thermotoga‐Fervidobacterium* (mTF) medium in a serum bottle (Wheaton, USA) sealed with a black butyl rubber stopper (Glasgeratebau OCHS GmbH, Germany) at 70°C under anaerobic conditions (Nam *et al.*, 2002). Briefly, the mTF medium was prepared as follows: the medium was flushed with N_2_ gas, sterilized by autoclaving at 121ºC for 20 min and then supplemented with 10 ml l^−1^ of a vitamin stock solution (Wolin *et al.*, 1964), 10 ml l^−1^ of a trace element stock solution (Balch *et al.*, 1979) and 3 ml l^−1^ of 25% (w/v) Na_2_S·9H_2_O solution (as a reducing agent) prior to use. Chicken feathers were washed with deionized water to remove unwanted materials such as skin and dust, air‐dried to remove moisture and then used as growth substrate.

To investigate the effects of carbon and nitrogen sources on *F. islandicum* AW‐1 growth, cells were grown in the mTF medium supplemented with 5 g l^−1^ glucose or 8 g l^−1^ native chicken feathers. Cells were centrifuged at 10 000 *g* for 30 min at 4ºC and resuspended in 0.2 µm of filtered saline. The cell concentrations were determined by measuring the absorbance at 600 nm using a spectrophotometer (A_600_ = 1.0 corresponds to 4.0 × 10^8^ cells ml^−1^) or direct cell counting using a Neubauer chamber (depth, 0.1 mm × area, 0.0025 mm^2^; Marienfeld, Germany) with a phase‐contrast microscope (Olympus BX43, Tokyo, Japan). The harvested cells were frozen with liquid nitrogen and stored at −80ºC until use. Unless otherwise noted, the growth experiments were performed at least in triplicate.

### Genome sequencing, assembly and annotation

Genome sequencing was performed using a single‐molecule real‐time (SMRT) sequencing platform on a PacBio RS II instrument (Pacific Biosciences, Menlo Park, CA, USA) with P6C4 chemistry (Pacific Biosciences) (Eid *et al.*, 2009). Genomic DNA was isolated from cells grown for 12 h (5–7 × 10^8^ cells ml^−1^) in mTF medium using a QIAamp DNA Mini Kit (Qiagen), as described previously (Lee *et al.*, 2015b). The generated sequencing reads were *de novo*‐assembled using the hierarchical genome assembly process (HGAP) (Chin *et al.*, 2013) protocol RS HGAP Assembly 2 in SMRT analysis version 2.3.0 (Pacific Biosciences). The coding sequences (CDSs) were predicted using the Prokaryotic Genome Annotation Pipeline (PGAP) software on NCBI. The genes in the assembled genome were annotated using NCBI clusters of orthologous gens (COG) (Tatusov *et al.*, 2000). The BLASTCLUST parameters for identifying internal clusters were ‘‐L .8 –b T –S 50’. Proteins with Pfam domains, signal peptides and transmembrane helices were identified using InProScan searches against HMMPfam (Bateman *et al.*, 2013), SignalPHMM (Petersen *et al.*, 2011) and TMHMM (Krogh *et al.*, 2001) via the Blast2Go service (Conesa *et al.*, 2005). Additional gene prediction and functional annotation were carried out using the eggNOG (evolutionary genealogy of genes: Non‐supervised Orthologous Groups) platform (Huerta‐Cepas *et al.*, 2016). Further classification of the identified proteases was carried out using the MEROPS peptidase DB (http://www.ebi.ac.uk/merops/). The complete *F. islandicum* AW‐1 genome sequence has been deposited in the DDBJ/EMBL/GenBank under the accession number NZ_CP014334.1.

### cDNA synthesis

Total RNA was isolated from bacterial cells by using the RNeasy Mini Kit (Qiagen, Hilden, Germany) according to the manufacturer’s instructions. Briefly, cells were ground in a mortar using liquid nitrogen, and genomic DNA was eliminated via treatment with RNase‐free DNase I. After RNA extraction, the RNA quantity and quality were determined via electrophoresis of total RNA and measurement of the absorbance at 260 nm. For the RT‐PCR, cDNA synthesis was performed using a SuperScript III Reverse Transcriptase Kit (Invitrogen, Carlsbad, USA). The reaction mixture (13 µl) contained 1 µg of total RNA, 4 µl of 2.5 mM dNTP (Takara, Kusatsu, Japan) and 300 ng of random primers (Invitrogen). After incubation at 65°C for 5 min, the mixture was rapidly chilled on ice. Reverse transcriptase (200 U; Invitrogen), 25 mM dithiothreitol (DTT, Sigma) and RNase inhibitor (40 U; Takara) were then added to the reaction mixture in a final volume of 20 µl. The reaction mixture was incubated at 25°C for 5 min and 50°C for 60 min, and then heated to 70°C for 15 min for inactivation. For the qRT‐PCR, RNA samples were prepared for the RT procedure using an iScript™ cDNA Synthesis Kit (Bio‐Rad, Hercules, USA). The reaction mixture (20 µl) containing 1 µg of total RNA was incubated at 25°C for 5 min for priming and 46°C for 20 min for RT, and then heated to 95°C for 1 min for inactivation.

### RT‐PCR

Most of the cDNA samples were diluted to a concentration of 25 ng µl^−1^. The primers used for RT‐PCR are listed in Table S2. The σ‐70 RNA polymerase subunit (RS04690) was used as a control. PCR was performed in a C1000 Touch™ Thermal Cycler (Bio‐Rad) using the following cycling conditions: 94°C for 5 min, 24 cycles at 98°C for 10 sec, 58°C for 30 sec, 72°C for 30 sec and a final extension at 72°C for 7 min. The PCR mixture (30 µl) contained 100 ng of cDNA, 10 pmol of each primer and 15 µl of EmeraldAmp GT PCR Master Mix (Takara). The PCR products were then loaded into a 2% agarose gel, electrophoresed and visualized under ultraviolet light after staining with ethidium bromide. Quantification of band intensity was performed with ImageJ (Schneider *et al.*, 2012). The amplification of a single product for all of the transcripts was confirmed via gel electrophoresis confirmed. The amplicons for all of the target sequences were gel‐purified and sequenced for target verification.

### qRT‐PCR

Most of the cDNA samples were diluted to a concentration of 10 ng µl^−1^ prior to PCR. qRT‐PCR amplification was performed using 2 × SsoAdvanced™ Universal SYBR Green Supermix including SYBR Green I (Bio‐Rad) with 20 ng of cDNA and 250 µM of each primer. The cycling conditions were as follows: an initialization step at 95°C for 30 sec, followed by 40 cycles of denaturation at 95°C for 10 sec and an annealing and extension step at 58°C for 30 sec. All of the qRT‐PCRs were performed in a CFX Connect™ Real‐Time System (Bio‐Rad). The primers used for qRT‐PCR are listed in Table S3. Standard curves were generated via linear regression of the resulting Ct values for serially diluted cDNA samples and used for efficiency estimates for each reaction according to the MIQE guidelines (Bustin *et al.*, 2009). All of the results were normalized to the expression level of the σ‐70 RNA polymerase subunit. The qRT‐PCR results were calculated using the $2^{-\Delta \Delta C_{t}}$ method (Livak and Schmittgen, 2001) in comparison with the σ‐70 subunit levels.

### Quantification of feather degradation products

The free amino acid concentrations in culture broth were determined using the ninhydrin assay (Rosen, 1957). After cultivation, 150 μl of 3% (w/v) ninhydrin solution (dissolved in 2‐methoxyethanol) and 150 μl of acetate‐cyanide buffer (1 ml of 10 mM KCN, 3.33 ml of NaOAc·glacial and 18 g of sodium acetate trihydrate per 50 ml, pH 5.0–5.2) were added to 30 μl of bacterial cell‐free supernatant, boiled for 15 min for colour development and stopped by cooling on ice. After addition of 660 μl of isopropyl alcohol–water diluent (1:1, v/v), the absorbance was measured at 570 nm. All measurements were performed in duplicate. During the enzymatic hydrolysis of feathers, the sulfhydryl group abundance in the reaction mixtures was monitored as described previously (Ellman, 1959). After cultivation, 100 μl of reaction buffer (1 M potassium phosphate buffer, pH 8.0), 20 μl of 5 mM DTNB solution and 600 μl of distilled water were added to 300 μl of bacterial cell‐free supernatant. After a 2 min of incubation at room temperature, the absorbance was measured at 412 nm. All measurements were performed in duplicate.

### Cloning and expression of putative keratinases

Each gene was amplified by PCR from *F. islandicum* AW‐1 genomic DNA with appropriate primers (Table S4). PCR was performed in a C1000 Touch™ Thermal Cycler (Bio‐Rad) using the following conditions: 98°C for 5 min, 30 cycles at 98°C for 30 sec, 55°C for 15 sec and 72°C for 1 min kb^−1^, and a final extension at 72°C for 10 min. The PCR mixture (50 µl) contained 100 ng of genomic DNA, 10 pmol of each primer, 0.2 mM dNTP mix, 10 µl of 5 × PrimeSTAR buffer (Takara) and 1.25 U of PrimeSTAR polymerase. The PCR products were cloned into pTOP Blunt V2, and the resulting constructs were transformed into *E. coli* DH5α. Transformants containing pTOP Blunt V2 vectors harbouring protease‐encoding genes were selected on Luria–Bertani (LB) plates containing ampicillin (100 µg ml^−1^). Plasmid DNA was isolated from transformants, digested with restriction enzymes and ligated into the pET‐28a (+) vector. The resulting plasmids were transformed into *E. coli* BL21 (DE3) cells, which were then grown at 37°C in LB broth containing kanamycin (50 µg ml^−1^) to an absorbance at 600 nm of 0.4–0.6. After induction with 0.2 mM isopropyl‐β‐D‐thiogalactopyranoside, the cells were grown overnight at 37°C before they were harvested by centrifugation at 10 000 *g* for 30 min at 4°C.

### Purification of putative keratinases

Centrifuged cells were resuspended in 20 mM Tris‐HCl buffer (containing 500 mM NaCl and 10 mM imidazole, pH 7.9) and disrupted by sonication on ice. The lysed cells were then centrifuged at 10 000 *g* for 20 min at 4°C. The supernatant was heated at 70°C for 30 min and then centrifuged at 10 000 *g* for 20 min to remove denatured *E. coli* proteins. The resulting supernatant was applied to a Ni^2+^‐affinity column equilibrated with the same buffer. His‐tagged proteins were eluted with 20 mM Tris‐HCl buffer (500 mM NaCl and 250 mM imidazole). The fractions were analysed by SDS‐PAGE and visualized with Coomassie blue.

### Keratinolytic activity assays

To assess the keratinolytic activity of the crude extracts and purified enzymes, we measured the increase in free amino acids using the ninhydrin assay as described above. For the preparation of crude extracts, *F. islandicum* AW‐1 cells grown in mTF medium containing 0.5% (w/v) glucose at 70°C for 12 h were transferred to freshly prepared mTF medium with 0.5% glucose. After 10 h of incubation, the cells were centrifuged at 10 000 *g* for 30 min at 4°C. The cell pellets were disrupted by sonication on ice. Cell debris was removed by centrifugation at 10 000 *g* for 20 min at 4°C, and the resulting supernatant, designated as AWCE, was used for the enzymatic assays. The reaction mixtures (3 ml) containing 0.0375 mg of AWCE (12.5 µg ml^−1^), 0.2% native feathers as a substrate, 50 mM Hepes buffer (pH 8.0), 10 mM DTT and 0.15 mg of purified enzyme (50 µg ml^−1^) were incubated at 72°C in Hungate tubes under anaerobic conditions. At each time point, 0.05 ml of reaction mixture was sampled for free amino acids.

## Conflict of interest

None declared.

## Supporting information


**Fig. S1**. Circular representation of the *F. islandicum* AW‐1 genome. From inside: The inner green‐ and outer dark brown‐colored histograms in the first track represent regions of AT and GC richness, respectively. The second track indicates the GC skew wherein sharp blue peaks and dark yellow peaks represent regions with positive and negative values, respectively. The multicolored histogram in the third track represents ORFs coded according to functional classification: blue rods, 47 tRNAs; red, 6 rRNAs. The fourth outer circle indicates protease loci in the genome to scale. The figure was generated with CL genomics 1.55 software (Chun Lab, Inc.).
**Fig. S2**. Protease families in the *F. islandicum* AW‐1 genome with number of protease‐encoding genes in each family indicated. Clan information was obtained based on the MEROPS database, and the families were classified using their conserved domains. Each family is identified by a letter representing the catalytic type of the member proteases together with a unique number. Aspartic (A), cysteine (C), metallo (M), serine (S), threonine (T), unknown (U). Most of the proteases belong to the M48, M42, and S14 families.
**Fig. S3.** Growth phase‐dependent expression levels of protease‐encoding genes. (a) For RT‐PCR, cells were grown in mTF medium supplemented with glucose or feathers. The quality of the mRNA was assessed via electrophoretic examination of the rRNA measurement of the absorbance at 260 nm using a spectrophotometer. (b) RT‐PCR for the expression profiling of *F. islandicum* AW‐1 protease‐encoding genes. The σ‐70 RNA polymerase subunit was used as a positive control for expression. BF, RNA extracted from harvested bacteria cells before yeast extract depletion; AF, RNA extracted from harvested bacteria cells after yeast extract depletion.
**Fig. S4**. SDS‐PAGE analysis of purified recombinant proteins using Ni^2+^‐affinity chromatography. (a) SDS‐PAGE analysis of whole‐cell extracts of transformants. Lane M, molecular weight marker; lane C, whole cell extract of *E. coli* BL21(DE3) with the expression vector; lane 1, peptidase M55 (encoded by RS10455, 31 kDa); lane 2, CPBP family intramembrane metalloprotease (RS08750, 46 kDa); lane 3, β‐aspartyl‐peptidase (RS08100, 42 kDa); lane 4, ATP‐dependent protease (ADP) (RS09910, 88 kDa); lane 5, insulinase family protein (RS01070, 44 kDa); lane 6, ATP‐dependent protease ATPase subunit HslU (RS01075, 51 kDa). (b) SDS‐PAGE analysis of the purified recombinant keratinases. Lane M, molecular weight marker; lane 1, peptidase M55 (encoded by RS10455, 31 kDa); lane 2, CPBP family intramembrane metalloprotease (RS08750, 46 kDa); lane 3, β‐aspartyl‐peptidase (RS08100, 42 kDa); lane 4, ATP‐dependent protease (RS09910, 88 kDa); lane 5, insulinase family protein (RS01070, 44 kDa); lane 6, ATP‐dependent protease ATPase subunit HslU (RS01075, 51 kDa).
**Table S1**. Genomic features of *F. islandicum* AW‐1.
**Table S2**. Primers used for RT‐PCR analysis.
**Table S3**. Primers used for qRT‐PCR analysis.
**Table S4**. Primers used for gene cloning.
