# Supplementary material for: Identification of keratinases from Fervidobacterium islandicum AW‐1 using dynamic gene expression profiling
Source: Microb Biotechnol. 2019 Oct 15;13(2):442–57. doi: 10.1111/1751-7915.13493 (PMC7017815; doi:10.1111/1751-7915.13493)
Supplement: Supplementary file 1 — Fig. S1. Circular representation of the F. islandicum AW‐1 genome. From inside: The inner green‐ and outer dark brown‐colored histograms in the first track represent regions of AT and GC richness, respectively. The second track indicates the GC skew wherein sharp blue peaks and dark yellow peaks represent regions with positive and negative values, respectively. The multicolored histogram in the third track represents ORFs coded according to functional classification: blue rods, 47 tRNAs; red, 6 rRNAs. The fourth outer circle indicates protease loci in the genome to scale. The figure was generated with CL genomics 1.55 software (Chun Lab, Inc.). Fig. S2. Protease families in the F. islandicum AW‐1 genome with number of protease‐encoding genes in each family indicated. Clan information was obtained based on the MEROPS database, and the families were classified using their conserved domains. Each family is identified by a letter representing the catalytic type of the member proteases together with a unique number. Aspartic (A), cysteine (C), metallo (M), serine (S), threonine (T), unknown (U). Most of the proteases belong to the M48, M42, and S14 families. Fig. S3. Growth phase‐dependent expression levels of protease‐encoding genes. (a) For RT‐PCR, cells were grown in mTF medium supplemented with glucose or feathers. The quality of the mRNA was assessed via electrophoretic examination of the rRNA measurement of the absorbance at 260 nm using a spectrophotometer. (b) RT‐PCR for the expression profiling of F. islandicum AW‐1 protease‐encoding genes. The σ‐70 RNA polymerase subunit was used as a positive control for expression. BF, RNA extracted from harvested bacteria cells before yeast extract depletion; AF, RNA extracted from harvested bacteria cells after yeast extract depletion. Fig. S4. SDS‐PAGE analysis of purified recombinant proteins using Ni2+‐affinity chromatography. (a) SDS‐PAGE analysis of whole‐cell extracts of transformants. Lane M, molecular weight marker; [file MBT2-13-442-s001.docx]

**Supporting information**

**Identification of keratinases from *Fervidobacterium islandicum* AW-1 using dynamic gene expression profiling**

Eunju Kang^1^, Hyeon-Su Jin^2^, Jae Won La^2^, Jae-Yoon Sung^2^, Soo-Young Park^2^, Won-Chan Kim^1^, and Dong-Woo Lee^2,*^

^1^School of Applied Biosciences, Kyungpook National University, Daegu 41566, South Korea.

^2^Department of Biotechnology, Yonsei University, Seoul 03722, South Korea.

**Sub-title:** Keratinases from *F. islandicum* AW-1

*To whom correspondence should be addressed:

**Dong-Woo Lee**, Department of Biotechnology, Yonsei University, Seoul 03722, South Korea.

Tel.: +82-2-2123-2886; Fax: +82-2-362-7265.; E-mail: [leehicam@yonsei.ac.kr](mailto:leehicam@yonsei.ac.kr)


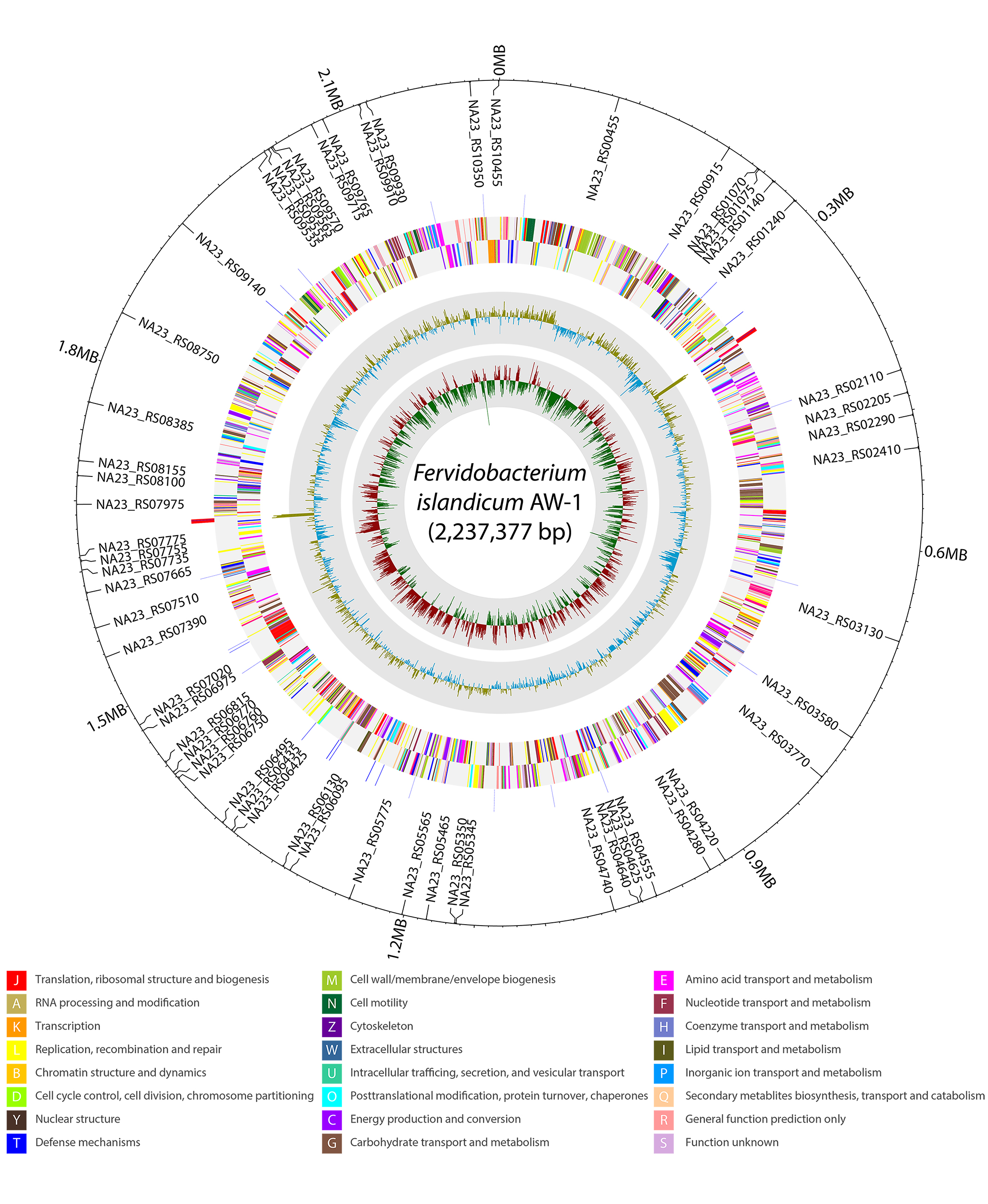


**Figure S1. Circular representation of the *F. islandicum* AW-1 genome.** From inside: The inner green- and outer dark brown-colored histograms in the first track represent regions of AT and GC richness, respectively. The second track indicates the GC skew wherein sharp blue peaks and dark yellow peaks represent regions with positive and negative values, respectively. The multicolored histogram in the third track represents ORFs coded according to functional classification: blue rods, 47 tRNAs; red, 6 rRNAs. The fourth outer circle indicates protease loci in the genome to scale. The figure was generated with CL genomics 1.55 software (Chun Lab, Inc.).


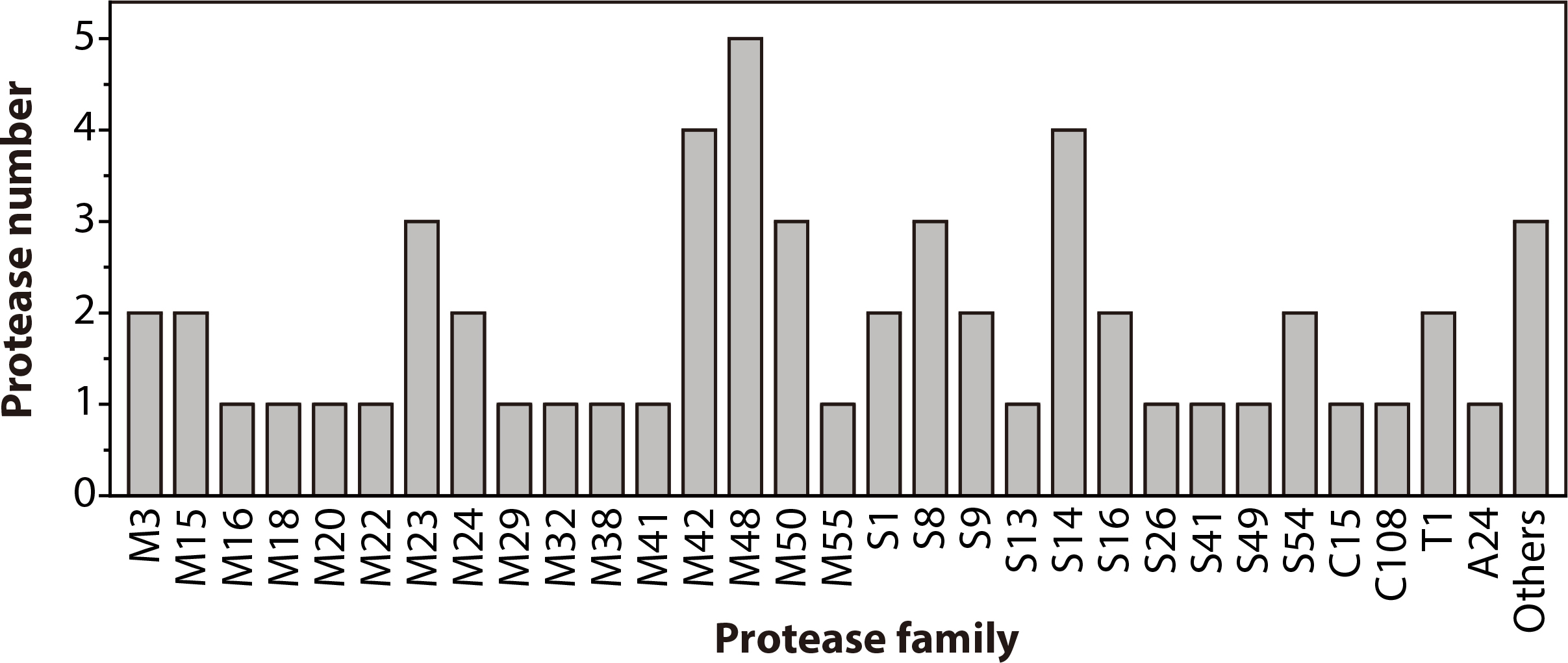


**Figure S2. Protease families in the *F. islandicum* AW-1 genome with number of protease-encoding genes in each family indicated.** Clan information was obtained based on the MEROPS database, and the families were classified using their conserved domains. Each family is identified by a letter representing the catalytic type of the member proteases together with a unique number. Aspartic (A), cysteine (C), metallo (M), serine (S), threonine (T), unknown (U). Most of the proteases belong to the M48, M42, and S14 families.

**
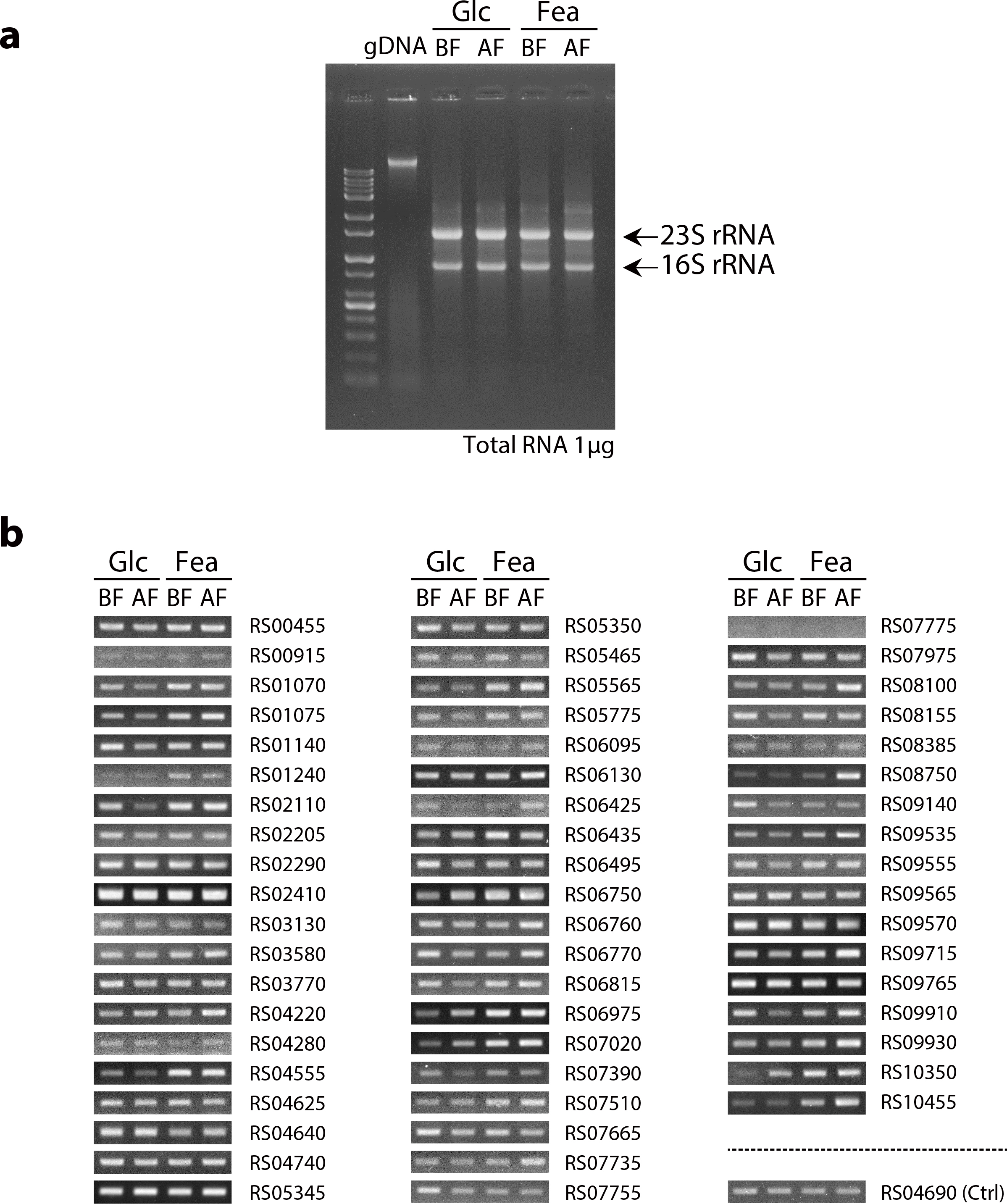
**

**Figure S3. Growth phase-dependent expression levels of protease-encoding genes.** (a) For RT-PCR, cells were grown in mTF medium supplemented with glucose or feathers. The quality of the mRNA was assessed via electrophoretic examination of the rRNA measurement of the absorbance at 260 nm using a spectrophotometer. (b) RT-PCR for the expression profiling of *F. islandicum* AW-1 protease-encoding genes. The σ-70 RNA polymerase subunit was used as a positive control for expression. BF, RNA extracted from harvested bacteria cells before yeast extract depletion; AF, RNA extracted from harvested bacteria cells after yeast extract depletion.

**
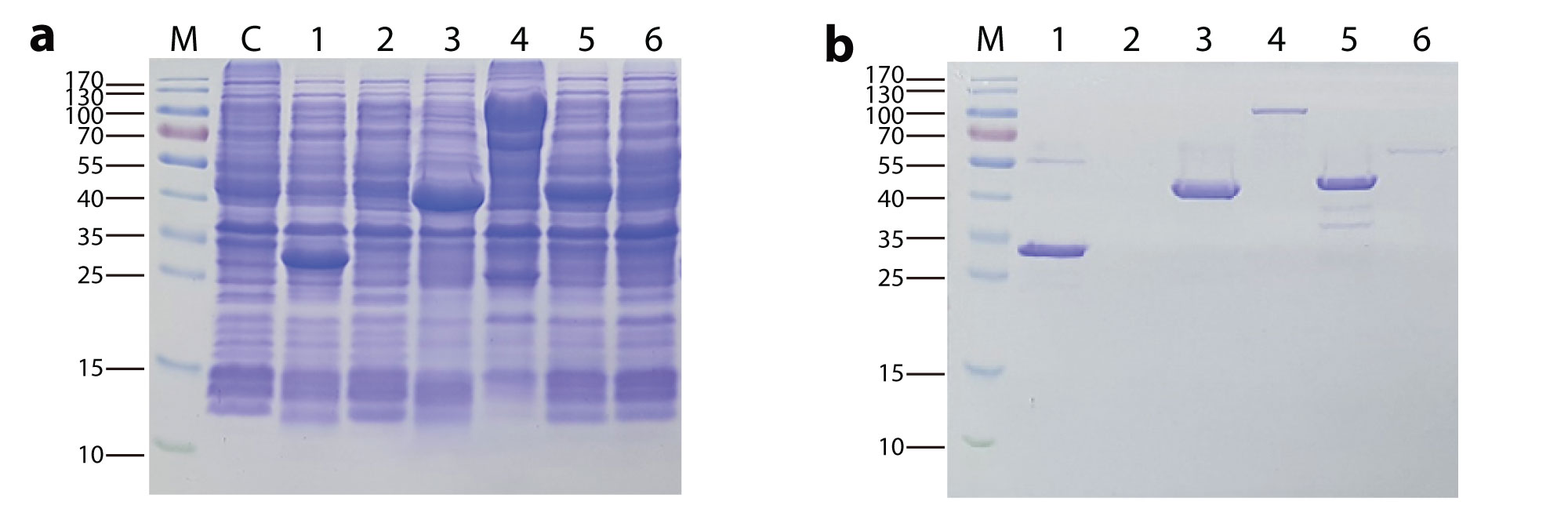
**

**Figure S4. SDS-PAGE analysis of purified recombinant proteins using Ni^2+^-affinity chromatography.** (**a**) SDS-PAGE analysis of whole-cell extracts of transformants. Lane M, molecular weight marker; lane C, whole cell extract of *E. coli* BL21(DE3) with the expression vector; lane 1, peptidase M55 (encoded by RS10455, 31 kDa); lane 2, CPBP family intramembrane metalloprotease (RS08750, 46 kDa); lane 3, β-aspartyl-peptidase (RS08100, 42 kDa); lane 4, ATP-dependent protease (ADP) (RS09910, 88 kDa); lane 5, insulinase family protein (RS01070, 44 kDa); lane 6, ATP-dependent protease ATPase subunit HslU (RS01075, 51 kDa). (**b**) SDS-PAGE analysis of the purified recombinant keratinases. Lane M, molecular weight marker; lane 1, peptidase M55 (encoded by RS10455, 31 kDa); lane 2, CPBP family intramembrane metalloprotease (RS08750, 46 kDa); lane 3, β-aspartyl-peptidase (RS08100, 42 kDa); lane 4, ATP-dependent protease (RS09910, 88 kDa); lane 5, insulinase family protein (RS01070, 44 kDa); lane 6, ATP-dependent protease ATPase subunit HslU (RS01075, 51 kDa).

**Table S1. Genomic features of F. islandicum AW-1**

| **Feature** | **Value** |
| --- | --- |
| Domain | Bacteria |
| Genome size (bp) | 2,237,377 |
| No. of contigs (with PEGs) | 1 |
| GC content (%) | 40.75 |
| Total number of genes | 2,065 |
| Protein coding genes (CDS) | 1,897 |
| rRNA genes (5S, 16S, 23S) | 6 (2, 2, 2) |
| tRNA genes | 47 |
| ncRNA | 3 |
| Pseudogenes | 112 |
| CRISPR arrays | 2 |
| GeneBank No. | NZ_CP014334.1 |

**Table S2. Primers used for RT-PCR analysis**

| **Gene name** | **Sequence (5'-3')** | **Tm (℃)** |
| --- | --- | --- |
| RS00455 | GCAAATTCACTGTAAGAAACGGATT | 57.9 |
|  | CATCATCGTTCACCTCCACTCTTA | 59.2 |
| RS00915 | ACTGGAGTTTGCGACAAGAAATAT | 58.0 |
|  | ACCTTCTTGTAGCTTATCCCCAT | 57.9 |
| RS01070 | CCTGAGTTCACAGACAGAAACGTTG | 58.7 |
|  | CCGTCTTAAACCCATCTGTCAGCT | 59.4 |
| RS01075 | GGGATTTGGTAGAGATAGCTGTGAA | 56.6 |
|  | GGCATTCCAACGAAACCTACG | 57.9 |
| RS01140 | AAAGGCCCAGGCTGAACTGC | 60.0 |
|  | ACCGACACCAGGAGGTCCAAC | 59.5 |
| RS01240 | GCGCTTGCAAGAAGATTCCAAGT | 60.5 |
|  | CCCATTATCTTACCAACGGGTATTC | 57.7 |
| RS02110 | GCCGGTTGATAGCTCGTATATAACG | 58.9 |
|  | AACTCTTCCCTTAGCATATTTGCCA | 58.3 |
| RS02205 | TCTATCCGAGGCAAATTGTTGATC | 58.2 |
|  | CTTGTGGAGCGTTGTAACTCA | 57.6 |
| RS02290 | AGTGCTCCATGTATAGTATTTATAGACG | 57.6 |
|  | TTTGTCTATTGGTTTATTTCTTAGGTGAAC | 58.0 |
| RS02410 | CCGCAAATGGCATTGGAGATA | 59.2 |
|  | GTTCTCGTTCTTTCAATTCCGCT | 57.5 |
| RS03130 | GAATTTGTCAGCGGAGAGGGA | 59.1 |
|  | GCCGCCAAGAAAAGTTCAAGTTA | 59.3 |
| RS03580 | TGAAAGATAAGCTCAACGACTGCG | 59.4 |
|  | TCAACAACAACAGGCTCACCTTT | 57.3 |
| RS03770 | GATCCTGTGTTGGTCCTTCCCG | 61.2 |
|  | CGCCATAACACCTTCGAATGCA | 61.4 |
| RS04220 | CGGAATGAAGGCAGAAGAAGTGG | 60.7 |
|  | GCCCAAATTCGTGTATCGTAGAGT | 57.3 |
| RS04280 | GTGCCGTTCCACCACACTTAAA | 58.3 |
|  | GAGTACGTGTGTGCAAGCGCC | 60.1 |
| RS04555 | ACGAACGTGCCCAGCAGATG | 60.4 |
|  | CCGTTCCTTCCTGAGATGCG | 59.0 |
| RS04625 | GGTGTGTTGCAGGTGTACCAAAC | 57.8 |
|  | GGAATATTCGGCAAGAACGGTACA | 59.9 |
| RS04640 | TTTCACCTTGAGGAATAACTATATCATCG | 58.4 |
|  | TTTGGTTCGGGCTTGGTAGTT | 59.1 |
| RS04740 | GGAGCAACGGTTATGAAAGGCA | 59.8 |
|  | CACCGGAACCTATTGCAGCTAT | 56.8 |
| RS05345 | GGACCAACTGGAAGTGGGAAGAC | 59.5 |
|  | CGTTGGCTATAGTTCCTTCCACG | 58.6 |
| RS05350 | GGTGGTCATACTCTAATACTAAAAACTG | 57.0 |
|  | AAGGTCACTTATAGGAAGCTCAGAA | 58.3 |
| RS05465 | GCATGGTGGATTTCTGCATTTG | 58.7 |
|  | GGATAAATCCCAGTGCGATGTTTAT | 58.2 |
| RS05565 | TGACATTGCCTTGCATTTGGG | 60.1 |
|  | CGTTACGTTTGAGCGGTGCA | 59.1 |
| RS05775 | GGTGACAACAATCCCACAGACC | 57.5 |
|  | GCAGCGCACACCAATGTAACG | 60.8 |
| RS06095 | GGGCGGACATCTTGCTTTGTTAG | 60.6 |
|  | CAAGAGCTTCGCCACCAGCC | 61.5 |
| RS06130 | GAGCTAAGGGCAGGAACAGCC | 58.8 |
|  | TCCCAAGACGTTCGTACAACACC | 59.6 |
| RS06425 | CCTTCGCGGCAAATGTAGACATT | 60.9 |
|  | CCAAGTGAAATCAGTGCACCAAG | 58.1 |
| RS06435 | ACACACAGTAACCGTTGGCGTTA | 58.0 |
|  | CGGTTTGGACGTAAACTCCAAGG | 60.4 |
| RS06495 | GAAACGCTGAACGAATAACGGTAA | 58.2 |
|  | CCTCCACTTTGAGCAGTTTATCCC | 58.9 |
| RS06750 | GATGATCCTGCACTTGTTGGTGG | 60.0 |
|  | CTGAAACTCCATCGCTTCTGCT | 57.2 |
| RS06760 | GGACGAAGTGGGCTTTCTGATTAC | 58.9 |
|  | GCTTTGGCGATTATGTAATCTCCC | 58.8 |
| RS06770 | CACCCATTTTTATTTTTGGATAGCCC | 58.5 |
|  | CATGATGAATCAAAACTGGAAGGAT | 56.9 |
| RS06815 | CGGTCAACCATGGTGCGAAA | 61.0 |
|  | TGTGGCATCGTTGAAATAATCG | 57.0 |
| RS06975 | CGAGCGTGCGTACGATATTTACTC | 58.8 |
|  | TTGTACCTGCGGCAAGTAGCAC | 58.8 |
| RS07020 | CGACGGTTTCCGTAAACGAAG | 58.1 |
|  | TGCCGATTCCGTGTCCTACAA | 59.7 |
| RS07390 | GGACGAGAAAGAACGACGAATAAG | 57.1 |
|  | CCCAACGTAAAGCTCCATTTAGAA | 57.4 |
| RS07510 | TGGTGGCTTGACAGGTCTTC | 58.9 |
|  | GGGTTGTTCAAATACGATGCCATAG | 59.5 |
| RS07665 | CATTCGAGCTGGAGGTTGACG | 59.1 |
|  | CACAGCAATAAGCCTATCAACAACC | 57.8 |
| RS07735 | TTCATTATTGTTCCACTGAGGTCTG | 58.1 |
|  | ATGTTGAAGATTTTGTTAGAGAGTGTTC | 57.7 |
| RS07755 | CCGACGAATATTCCAACGTAGAGT | 57.3 |
|  | CTGTTGCCGATGCTCGCATC | 61.0 |
| RS07775 | GAATTCTTGTCGAACGGTTTGTTG | 58.5 |
|  | CCAAAGCTGGTAATCATTCCAAACC | 60.5 |
| RS07975 | GCATGACGGGAGCACAGCTT | 59.6 |
|  | GAAGAACACCCATGCGAGGTTT | 58.8 |
| RS08100 | GTCATCGGTGTTGGCGAAATC | 58.7 |
|  | CGGTTAGGTCGATTGTTCCACC | 59.0 |
| RS08155 | GAAGGCGTAAGGGAGATAGAAGGT | 57.1 |
|  | GGGCAGACTTGATCACTATGAACTC | 57.1 |
| RS08385 | TTCCACCTTCGTTATTGAGCAAA | 57.3 |
|  | ATCAAGGATGGCACGAATCTTTG | 58.8 |
| RS08750 | TCTTTTGTTTCGTTCTTTTTAGTTTTGTAC | 57.9 |
|  | TGTATGCCATAAACACCGAAAAGAAA | 59.0 |
| RS09140 | AAGGAGTCTTTGAATTTGAAATCTGAC | 57.7 |
|  | TTTATTATCATTCCAATTCCTTTTCAGC | 56.5 |
| RS09535 | CCCAGTCTTACAAATCTTCACGCA | 59.0 |
|  | AGGGATTTCCACTATGAAAGGCAG | 58.5 |
| RS09555 | AATGCTATGATGGACGCAGTCAA | 58.1 |
|  | TTCACAGAAGAGCAGAATGCCC | 57.9 |
| RS09565 | CGTGTCTTACGTCAACGGTGGA | 59.3 |
|  | CCGTGAGTACCTCACTTCCAATCT | 57.3 |
| RS09570 | GGCGTATTCGTAACAGACAACAGG | 58.5 |
|  | GTTGTCCAAGTTTGCCGGCA | 60.2 |
| RS09715 | GGGCGAAACACCAGAAGAACTC | 58.6 |
|  | TATCGTATCCCGCAACGTGTG | 57.9 |
| RS09765 | ACGAGCCTGTCCCTTCAGAACTT | 59.1 |
|  | CCGCCCTGTTGTTCTCTTTCAGA | 61.2 |
| RS09910 | ACCCGGCGCTTTGCATAAAG | 60.7 |
|  | CCAGGTCCGAAATGTTCTCAACA | 59.6 |
| RS09930 | CGTTAACCCTTTCTGATTTGGCG | 60.4 |
|  | CCATCCAGCGAATTTAACTACCC | 57.5 |
| RS10350 | GCGCACTTCAAAGTACGGATCC | 59.4 |
|  | GCGTAGATGTCCATACCGATGG | 57.8 |
| RS10455 | TGCAGCTGGTGACAACATACCC | 59.3 |
|  | CCCAACTAAGCATCCAAGAGGAA | 58.1 |
| RS04690 (RNA polymerase subunit σ-70/Ctrl) | GGGTTCTGATGACATTGATGATGT | 56.3 |
|  | CGGGACTTAACTTATCCAACGC | 56.5 |

**Table S3. Primers used for qRT-PCR analysis**

| **Gene name** | **Sequence (5'-3')** | **Tm (℃)** |
| --- | --- | --- |
| RS01070 | ATAACAAGAGAAGATTTGGAACGATTC | 57.3 |
|  | GTTTCAAGTCTTTCTCATCAACGT | 57.0 |
| RS01075 | GAAAGAGGGCAGAGCTAAGGG | 59.2 |
|  | GGCATTCCAACGAAACCTACG | 57.9 |
| RS02110 | CCGTTGTTTTGGAACATCCTGA | 58.4 |
|  | AACTCTTCCCTTAGCATATTTGCCA | 58.3 |
| RS03580 | GAAATTACTGGGAGCGGATGATAT | 57.5 |
|  | TCAACAACAACAGGCTCACCTTT | 57.3 |
| RS04555 | CCCGAACATTTGAAGAGTGTTAAC | 57.6 |
|  | CCGTTCCTTCCTGAGATGCG | 59.0 |
| RS05565 | GCGGTTGCCGAGTTATTGAAG | 59.3 |
|  | CGTTACGTTTGAGCGGTGCA | 59.1 |
| RS05775 | CTGGAATCAGATGGGCAGTAGAC | 59.5 |
|  | GCAGCGCACACCAATGTAACG | 60.8 |
| RS06425 | CTCTGTTCACAGATTTCAAGCTCA | 58.3 |
|  | CCAAGTGAAATCAGTGCACCAAG | 58.1 |
| RS08100 | CCAATCCAACATCTGTATCCTACAC | 58.3 |
|  | CGGTTAGGTCGATTGTTCCACC | 59.0 |
| RS08155 | CGATACAACTGCTGTAGCTTTGAAC | 59.7 |
|  | GGGCAGACTTGATCACTATGAACTC | 57.1 |
| RS08750 | GCAATGGTGTTATCTTCAATCTCAGC | 60.0 |
|  | ACGCTCACGTACGTTTGAATATTG | 59.2 |
| RS09535 | GTTCTCCCCTTACCCGATGGTA | 60.0 |
|  | AGGGATTTCCACTATGAAAGGCAG | 58.5 |
| RS09765 | ACGAGCCTGTCCCTTCAGAACTT | 59.1 |
|  | TGATTGCTACGTCGAGTAATCCAC | 59.8 |
| RS09910 | CCCCGAGATATACGAACTACTGC | 59.2 |
|  | CCAGGTCCGAAATGTTCTCAACA | 59.6 |
| RS10350 | CAAAATCCCAGTCGATGACCC | 58.0 |
|  | GCGTAGATGTCCATACCGATGG | 57.8 |
| RS10455 | ATGGACCACACATATTCGAGTTCA | 59.1 |
|  | CCCAACTAAGCATCCAAGAGGAA | 58.1 |
| RS04690 (RNA polymerase subunit σ-70/Ctrl) | GGGTTCTGATGACATTGATGATGT | 56.3 |
|  | TATCCACGTTGACAATTTAGAATCTC | 56.6 |

**Table S4. Primers used for gene cloning**

| **Gene name** | **Sequence (5'-3')** | **Tm (℃)** |
| --- | --- | --- |
| RS01070 | CATATGCTTAAACTTGGAGAGAATATATACTAC | 57.7 |
|  | GGATCCCTAAACATAAATATCTGGGAG | 57.9 |
| RS01075 | GGATCCTTGACGAATTTTGACAATTTGAC | 61.1 |
|  | CTCGAGTCAAAGGATGTAAGCTGCT | 61.9 |
| RS08100 | GCTAGCATGATAAAAATTATAAAGAACG | 55.5 |
|  | CTCGAGTCAAAATTCAAAGTTTAAGTTC | 57.4 |
| RS08750 | GGATCCATGAAACAGTCAAAGGATAGTTTT | 61.2 |
|  | CTCGAGTTAGCAGGCATCCTGGGAG | 65.4 |
| RS09910 | GGATCCATGCCTTTGAAGAAGATTAGGGT | 63.7 |
|  | CTCGAGTTACATTTTTTCGCGCTTTTTCTT | 63.0 |
| RS10455 | CATATGATGGCTACAACAAGAAAGAAAA | 57.9 |
|  | GGATCCTTACACTAAAATCTTAGCTGCTGC | 60.1 |
